# Comparative performance analysis of different microfilaria testing methods for *Dirofilaria immitis* in canine blood

**DOI:** 10.1186/s13071-024-06537-6

**Published:** 2024-11-11

**Authors:** Rachel C. Smith, Trey D. Tomlinson, Joy V. Bowles, Lindsay A. Starkey

**Affiliations:** 1 Department of Veterinary Pathobiology, Oklahoma State University College of Veterinary Medicine, Stillwater, OK USA; 2 Kansas State University College of Veterinary Medicine, Manhattan, KS USA; 3 Department of Pathobiology, Auburn University College of Veterinary Medicine, Auburn, AL USA

**Keywords:** *Dirofilaria immitis*, Heartworm, Microfilaria testing, Comparative analysis, Canine

## Abstract

**Background:**

Microfilaria (MF) testing is an essential part of canine heartworm diagnostics, and it is recommended by the American Heartworm Society that a MF test be performed in tandem with antigen testing on every dog, every year, regardless of prevention status or history. There are a variety of methods that can be used to detect MF in canine whole blood; however, these methods widely vary in their sensitivities as well as practical factors, including time investment and cost. Additionally, some MF tests offer the advantage of being quantitative or allowing for morphological or molecular species identification, while other tests should only be used qualitatively.

**Methods:**

The purpose of this study is to evaluate the quantitative and qualitative performance of MF tests, including the 20 μL count, wet mount, 9 μL and 40 μL hematocrit tubes, thin smear, thick smear, modified Knott test (MKT), and conventional polymerase chain reaction (PCR).

**Results:**

Qualitatively, there was little difference in the performance of the 20 μL count, wet mount, MKT, and PCR. The MKT and PCR are the optimal MF tests, as these perform most reliably for detecting positives even when the MF per milliliter is relatively low, and in most cases, these two methods also allow for species-level confirmation of the identity. However, PCR tends to be a very costly test, and both PCR and MKT require a greater degree of expertise and time investment to perform than other tests. Even the lowest performance tests, including the thin smear and hematocrit tube methods, can reliably detect MF at very high burdens; although, caution should be advised when using low reliability methods, since there is a greater likelihood of failing to identify MF-positive dogs.

**Conclusions:**

Microfilaria (MF) testing is an essential part of heartworm diagnosis and screening in dogs, and test selection should balance practical factors such as cost and time investment with the patient’s risk of infection based on prevention status and history, clinical signs, and antigen testing results. This approach to MF testing will help minimize cost while avoiding failure to detect MF in infected dogs, especially when MF burden is low.

**Graphical Abstract:**

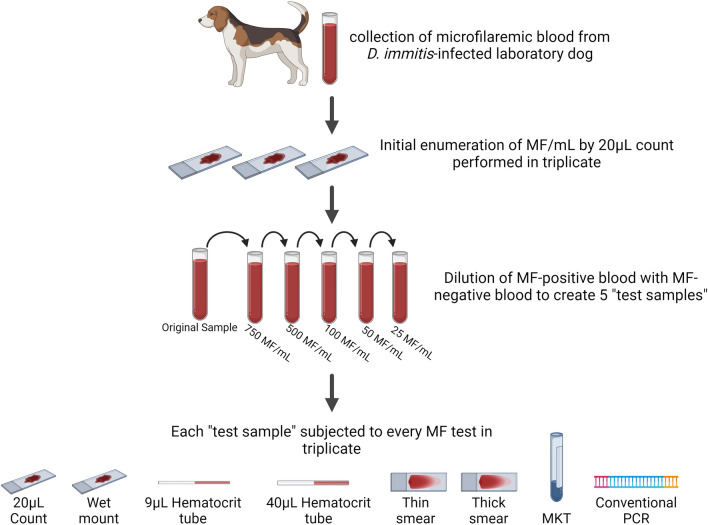

**Supplementary Information:**

The online version contains supplementary material available at 10.1186/s13071-024-06537-6.

## Background

*Dirofilaria immitis* is a mosquito-borne nematode that has worldwide notoriety for causing canine heartworm disease. It is well known that many mammalian vertebrates, including humans, may serve as hosts for *D. immitis*, although canids, both wild and domestic, contribute the most to the maintenance and propagation of the parasite due to their ability to support higher numbers of worms within the pulmonary arteries, as well as MF, which circulate within the peripheral blood [1, 2]. To briefly summarize the lifecycle of *D. immitis* in canine hosts, third-stage larvae (L3s) are deposited in a pool of hemolymph onto the skin during the bloodmeal by infected mosquitos. Subsequently, L3s enter the skin through the bite wound and begin migrating through the subcutaneous tissues, molting two additional times before reaching the pulmonary arteries. Immature adult parasites reach the pulmonary arteries approximately 70–110 days post infection (PI), where they will then complete sexual maturation [2]. Assuming at least two sexually mature worms of the opposite sex are in adequate proximity within the host, they can reproduce, resulting in microfilaria (MF), which circulate in the peripheral blood and become detectable 6–9 months PI [2]. Circulating MF may be ingested by female mosquitoes during blood feeding, and within a competent mosquito species, the parasite develops through the L1, L2, and, finally transmissible, L3 stage in 10–14 days, depending on temperature and humidity [2]. In the canine host, initial infection with larval stage heartworms usually progresses subclinically. However, once worms reach the pulmonary arteries, pathology inevitably develops within these vessels. Adult heartworms induce endothelial hyperplasia of the pulmonary arterioles, with lesions becoming more severe and extensive over time. Eventually, the vessels can become obstructed, resulting in pulmonary hypertension, which can lead to congestive heart failure [2]. In the absence of appropriate treatment, canine heartworm infection can become a fatal condition.

Heartworm disease is almost entirely preventable through the compliant use of macrocyclic lactone-based prophylactics, of which there are oral, topical, and injectable formulations for dogs [3]. Despite this, there remains substantial need for diagnostic tests, both for routine screening and clinicopathologic diagnosis. Currently, the American Heartworm Society advises that every dog, regardless of current prevention status or history, be tested with both an antigen (Ag) detection test and a MF test at least once every year [4]. Ag detection relies on detection of Ag circulating in the blood, which is primarily secreted by sexually mature female worms and becomes routinely detectable in dogs 5–7 months PI. A positive Ag test and MF test are highly indicative of patent infection with *D. immitis*; however, there are some scenarios in which Ag and MF testing results are seemingly discordant. In these cases, performing both tests simultaneously provides a more complete diagnostic picture, thus, why tandem use of these diagnostic tests is now the standard recommendation for annual screening for dogs [4]. For example, when Ag testing is positive and no MF are detected, this likely indicates that infection with *D. immitis* has not yet reached patency, worm numbers and spatial distribution are inadequate to allow for successful reproduction, or there is clearance of MF secondary to the immune system or use of a chemoprophylactic agent. A more puzzling situation occurs when no Ag is detected and MF testing is positive. This could indicate that the MF belong to a parasite species other than *D. immitis* or the infection is truly *D. immitis*, but antigen detection failed due to issues with the antigen test (e.g., improper processing, storage, or selection of an antigen test with a lower reported sensitivity) or, more importantly, an antigen forming an immune complex with host antibodies. There are several options available for MF recovery, and each test carries certain diagnostic and practical advantages and limitations. The modified Knott’s test (MKT) is the preferred MF test among parasitologists in most cases because it includes a concentration step and, therefore, has greater sensitivity, provides quantitative estimation of MF/mL burden, and allows one to morphologically confirm the identity of MF [4]. Additionally, the MKT requires little specialized equipment and can be performed relatively quickly within the clinic. Despite the ease with which an MKT can be performed, many clinics rely on other MF tests, such as wet mounts or visualization of MF following a hematocrit test. These methods require fewer supplies, less time, and very little blood volume and so are more cost effective and can easily be combined with other blood diagnostics. However, because these methods do not involve a concentration step and vary in the volume being used, they have substantially reduced sensitivity and are not reliably quantitative nor do they allow for confirmation of species identification. PCR-based diagnostics are now becoming more accessible to practicing veterinarians via availability through reference laboratories and provide the advantage of both high sensitivity and specificity. However, they are often the most expensive when compared with more classical diagnostic tests and require longer “turn-around” time due to needing to send the sample to a reference laboratory.

The objective of this study was to compare the performance of different MF test methods which are available for use by veterinarians in-clinic, are available at reference laboratories, or are performed in research settings. Each microfilaria test was performed on the same set of samples of canine blood which had known concentrations of MF per mL (MF/mL). Results were evaluated on the basis of (1) qualitative performance assessed by the ability of each test to positively recover or detect microfilariae (e.g. positive, unreliable, or negative) and (2) quantitative performance assessed by simple box plot to demonstrate the variance among test replicates and compare MF burden estimated by replicate to the originally calculated sample concentration.

## Methods

### Blood samples

Canine whole blood samples containing *D. immitis* MF were supplied by the Filariasis Research Reagent Resource Center (FR3) and collected from three purpose-bred laboratory dogs infected only with *D. immitis*. Microfilaria-negative canine whole blood to be used for dilution of MF-positive blood was also supplied by FR3 from a dog with no history of or potential for parasitic infection, including *D. immitis*. Blood samples were collected and transported via overnight ground shipping on ice to the Auburn University College of Veterinary Medicine then maintained at room temperature while awaiting processing. Performance of MF tests which depend on or are enhanced by motility of MF (20 μL, wet mount, and hematocrit tests) were prioritized, and were performed within approximately 24 h of receiving samples at Auburn University. All MF tests were performed within 48 h of sample receipt with the exception of PCR, for which aliquots of blood were stored at −20 °C until processing. Upon receipt, MF-positive blood samples were pooled into a single microfilaremic sample, which was then subjected to an initial MF quantification by performing the 20-μL count method described below in triplicate, and then, these replicates were averaged to determine the number of MF/mL for this undiluted blood sample. This method has been the preferred quantification method utilized by the Auburn University personnel when examining known microfilaremic samples in a research setting. Five “test” samples were created by diluting the MF-positive blood with MF-negative blood such that their concentrations were approximated to be 750, 500, 100, 50, and 25 MF/mL. These test samples were then subjected to each of the following test methods described below in triplicate. Three diagnostic parasitology experts were responsible for performing the techniques, reading all slides and quantifying microfilaria, and each diagnostic expert was responsible for reading one replicate for each test sample and MF test combination.

### A 20 μL count

A calibrated pipette was used to transfer 20 μL of sample to a glass slide and topped with a coverslip. The slides were examined with a compound microscope at low magnification, and MF were visually quantified. The quantity of MF was then multiplied by a factor of 50 to approximate MF per milliliter.

### Wet mount

Wet mounts were prepared according to a previously described protocol [5]. A disposable pipette was used to transfer one drop (approximately 50 μL) of sample to a glass slide and topped with a coverslip. The slides were examined with a compound microscope at low magnification and MF were visually quantified. The quantity of MF was then multiplied by a factor of 20 to approximate MF per milliliter.

### Hematocrit test

Hematocrit tests were prepared according to a previously described protocol [5]. Two sizes of hematocrit tubes, 9 μL and 40 μL, were filled with sample to approximately 75% capacity and packed with clay at one end. The filled tubes were then centrifuged at 16,000 rpm (13,700 rcf) for 2 min for clear separation of the plasma, buffy coat, and erythrocyte layers. The space directly above the buffy coat was visually evaluated with a compound microscope at low magnification for the presence of MF. The quantity of microfilaria was then multiplied by a factor of 111.11 and 25 for the 9 μL and 40 μL tubes, respectively, to approximate MF per milliliter.

### Thin smear

A calibrated pipette was used to transfer 4 μL of sample near the end of a glass slide. A second slide was placed at a 30–45° angle, slightly touching the drop of blood, and then pushed outward to smear the blood across the slide. The slides were allowed to dry at room temperature, fixed with absolute methanol, and then stained with Giemsa stain. The slides were examined with a compound microscope at low magnification, and MF were visually quantified.

### Thick smear

Thick smears were prepared according to the standard operating procedure from the NIH/NAID Filariasis Research Reagent Center (FR3). A disposable pipette was used to transfer one drop (approximately 50 μL) of water to a glass slide. A calibrated pipette was used to transfer 20 μL of sample to the slide and mix the sample with the water. The blood and water mixture was then spread evenly over a 15 mm × 25 mm area at the center of the slide using a toothpick and allowed to dry at room temperature overnight. The slides were then warmed to 50 °C for 1 h. The diluted Giemsa stain was carefully pipetted over the top of the slide, and excess stain was flushed with deionized water. The slides were allowed to dry completely at room temperature, then were examined with a compound microscope at low magnification, and MF were visually quantified. The quantity of MF was then multiplied by a factor of 50 to approximate MF per milliliter.

### Modified Knott test (MKT)

Modified Knott testing was performed according to a previously described protocol [5]. One milliliter of blood was transferred to a 15-mL centrifuge tube and mixed with 9 mL 2% formalin. The mixture was inverted several times then centrifuged at 1409*g* for 5 min. The supernatant was then discarded, one drop of methylene blue was added to the sediment, and the entire stained sediment was transferred to a glass slide and topped with a coverslip. The slides were examined with a compound microscope at low magnification, and MF were visually quantified as MF/mL.

### Polymerase chain reaction (PCR)

Prior to DNA extraction, the samples were stored at −20 °C. Genomic DNA was extracted from a 200 μL aliquot of each sample with the Cytiva Blood genomicPrep Mini Spin Kit (Cytiva, Marlborough, MA) according to the manufacturer’s protocol. A previously published set of primers (Fila 12SF: 5′-CGGGAGTAAAGTTTTGTTTAAACCG-3′ and Fila 12SR: 5′-CATTGACGGATGGTTTGTACCAC-3′) were used to amplify a 12S gene fragment (~330 bp) [6]. PCR reaction mixtures, amplification conditions, gel electrophoresis, and amplicon purification were performed as previously described [7]. Since these samples were from experimentally infected dogs, only select positive amplicons were sent for Sanger sequencing at the Oklahoma State University Molecular Core Facility (Stillwater, OK), and sequences were analyzed by BLAST and against the GenBank database to confirm the genetic identity.

## Results

Qualitative performance was assessed on the ability of the tests to recover or detect MF. Tests were considered positive if the test recovered MF across all replicates of a given sample, unreliable if at least one replicate of the test failed to recover MF for a given sample, and negative if no replicate recovered MF for a given sample (Table 1). Based on this assessment, the MKT and PCR tests had the best qualitative performance, as these tests positively recovered MF across all samples and replicates. Following in rank of performance were the 20 μL and wet mount, which positively recovered MF in the four most concentrated samples; the thick smear, which positively recovered MF in the three most concentrated samples; the 40-μL HCT tube and the thin smear, which positively recovered MF in the two most concentrated samples; and, lastly, the 9-μL HCT tube, which positively recovered MF only in the highest concentration sample.

**Fig. 1:**
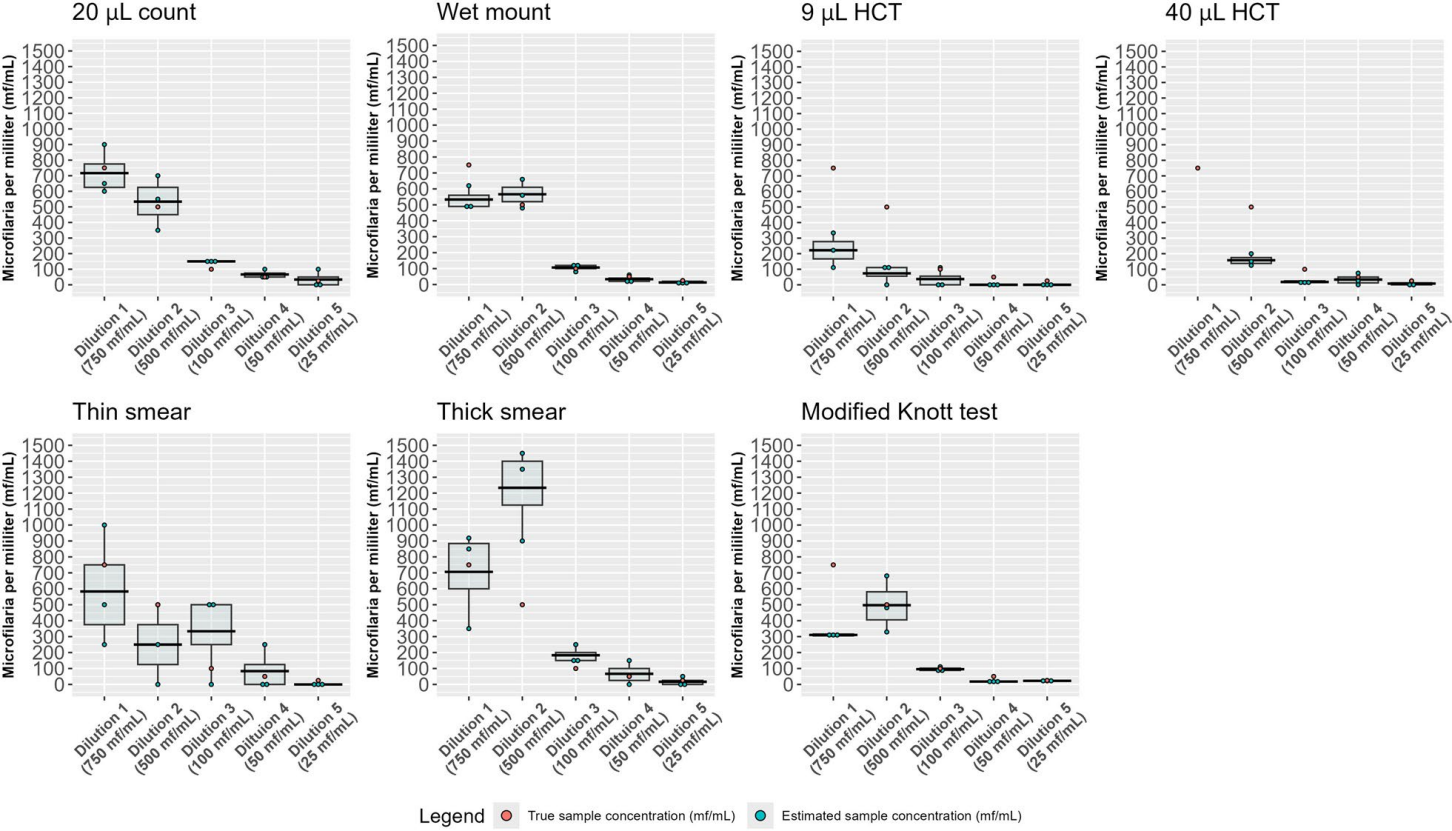
Comparison of microfilaria test performance for the detection or recovery of *D. immitis* in canine blood using boxplots to visually demonstrate variance between replicates

Quantitative performance was assessed by using a multiplication factor relative to the blood volume used for each test to estimate the total quantity of MF per milliliter for each sample. These estimates were then compared with the calculated concentration of each sample by dilution. Quantitative assessment has been depicted graphically for each test by box plots, with originally calculated dilution estimates and replicate estimates indicated by color-coded points (Fig. 1) (complete data provided in Additional file 1). Variation among replicates tended to be higher with greater MF burden in the samples, as indicated by a wider box plot. As the concentration of MF decreased, variation among replicates also decreased. Both the 9-μL and 40-μL HCT tube methods consistently underestimated the burden of MF for all samples; however, these methods also demonstrated less variation among replicates relative to other tests. The MKT demonstrated very little variation among replicates for each sample with the exception of the 500 MF/mL dilution; however, there was obvious underestimation of MF burden for the 750 MF/mL sample across all three replicates. A similar underestimation was present with the wet mount technique at that dilution. It is unclear why this underestimation occurred, as this does not fit the trend for the performance of the MKT or wet mount on other samples.

**Table 1:**
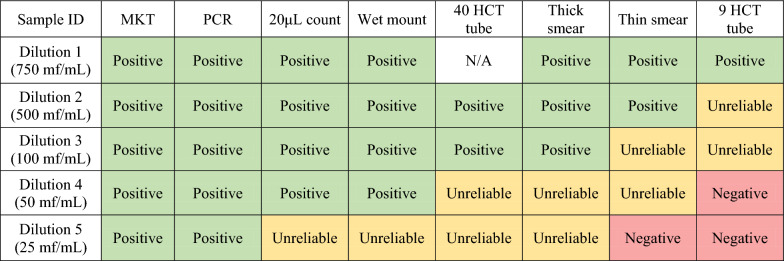
Color-coded comparative analysis of the qualitative performance of microfilaria tests for the detection or recovery of *D. immitis* in canine blood

Test performance is categorized as: positive, indicated in green—the test recovered or detected microfilariae for all replicates; unreliable, indicated in yellow—the test failed to recover or detect microfilariae on at least one replicate; or negative, indicated in red—the test failed to recover or detect microfilariae on all replicates. N/A indicates that the test was not performed for this dilution

## Discussion

In this study, the qualitative and quantitative performance of testing methods, which are commonly employed in-clinics, reference labs, or research settings, were analyzed and compared. We found that the MKT and PCR proved to be the most qualitatively reliable, particularly at low MF concentrations, and these were the only methods that reliably detected MF, even at a concentration of 25 MF/mL. When the blood concentration of MF was at least 50 MF/mL, the wet mount and 20 μL count methods had qualitative performance, which was equivalent with the MKT and PCR. Unsurprisingly, we observed that the qualitative reliability of MF testing appears to directly correlate to the blood volume input of the test. Concentration of greater blood volume into a single MF test increases the likelihood of MF recovery. As expected, we found that not all MF testing methods are suitable for quantifying MF burdens. In addition to having less qualitative reliability, lower blood volume tests, including the hematocrit tube method and the thin smear, often underestimated the burden of microfilaria; notably, use of a larger volume hematocrit tube resulted in higher sensitivity for MF detection in this study, and it would be expected that using a larger blood volume when preparing the thin smear would have a similar effect on sensitivity as long as the increase in blood volume did not compromise thin smear quality.

There are some inherent limitations of the present study that must be addressed. In routine practice, MF testing is typically performed directly following sample collection. Some MF testing methods, including the wet mount, 20 μL count, and hematocrit tube methods, are dependent on or enhanced by looking for motile MF. Although MF can survive temporarily ex vivo in a collected sample, death of MF within the sample over time likely reduces the sensitivity of motility-dependent MF tests. Because the MF-positive dogs were housed at a different site than the institution where testing and analysis were performed, efforts were taken to minimize the amount of time and temperature fluctuations between sample collection and MF testing, especially for those tests that are more reliant on MF motility. However, these present study conditions are not as ideal as performing MF testing immediately following sample collection. Another limitation is that the method used to quantify MF in the original sample was the 20 μL count performed in triplicate and averaged. Although the results of this study revealed that this method is less reliable when MF are at low concertation, the original samples had relatively moderate-to-high concentrations of MF per milliliter (1850–2717 MF/mL), and this method of quantitation is the research method of choice employed by the Auburn University Parasitology Research Lab. Should this study be replicated, it may be more accurate to perform initial MF quantification by performing the MKT method in triplicate in addition to a dilution method for quantification (i.e., 20 μL count); however, we do not feel that this methodology choice substantially impacted the findings of the present study. Furthermore, with relatively high MF counts, the chance for introducing human error with counting is higher, as MF crowding and overlapping would interfere with accurate visualization and delineation. Historically, filtration MF tests, such as the Difil, have been commonly used for recovery of MF. The authors of this study opted not to include this MF test method for analysis because the test apparatus and filters are no longer commercially available for purchase as a diagnostic kit.

Some other studies have compared the performance of select MF tests. In a study examining 100 shelter dogs from Florida for heartworm infection, 17 dogs were MF-positive by wet mount and MKT, with MF counts ranging from 3 to 43,280 MF/mL. MKT evaluation further revealed one additional dog to be MF-positive (13 MF/mL), suggesting that the MKT only slightly enhances diagnosis of MF compared with the wet mount [8]. Our study also found the MKT to be more reliable than the wet mount method, in that the MKT positively detected MF even at the lowest MF concentration (25 MF/mL), while the wet mount method unreliably recovered MF at this concentration. One should consider the possibility that because this previous study surveyed shelter dogs, which are at high-risk for heartworm infection, that the MF burden was high enough that the reliability of the MF test was less critical for detecting positives. That is to say that the difference in performance between the MKT and wet mount was less drastic because the sample population was more likely to have high burdens of MF, under which conditions the reliability of the MF test used is of less consequence. Another study comparing direct smear (wet mount) to MKT determined that the wet mount overall detected roughly 20% fewer infections compared with MKT [9]. In that study, the sample population was experimentally infected dogs, and all samples with > 50 MF/mL were detected by both methods; however, in samples with < 50 MF/mL, all were positive by MKT, but only 44% of the samples were positive by wet mount [9]. Similarly, we observed that at a minimum concentration of 50 MF/mL, both the wet mount and 20μL count methods have qualitative performance equivalent to the MKT, but when the concentration falls below 50 MF/mL, the wet mount and 20μL count methods become unreliable.

There are multiple factors that veterinarians must consider when choosing a MF test. Certainly practical matters such as cost of the test, time required to run the test, and the need for specialized equipment are all important factors in the clinic setting. However, it is also important to consider the circumstances in which the test is being performed and what additional information is offered by some diagnostic tests beyond simply recovery of MF. Microfilariae tests, which involve a concentration of larger blood volume into a single test, such as the MKT and PCR, will always provide a more reliable result than those that utilize very low blood volume. Additionally, both of these test options offer the advantage of species-level identification. The MKT can be run without specialized equipment, is inexpensive, and is accurately quantitative. However, distinguishing MF of different species relies on the expertise of the individual interpreting the slide as well as the sample quality. PCR tends to be highly sensitive and overcomes some of the pitfalls of the MKT because it does not rely on the morphological expertise of an individual reading a slide. However, PCR also tends to be the most expensive MF test, has an increased turn-around time for results, and is not yet available for in clinic testing. Microfilaria testing is an essential aspect for accurate screening and diagnosis of canine heartworm infection, helps to identify reservoirs of infection, and also aids in monitoring for presence of macrocyclic lactone-resistant *D. immitis* during and after treatment. Furthermore, performing MF tests that are capable of species identification can help to identify new and emerging filariids and prevent misdiagnosis of these infections as *D. immitis* [11]. Selection of a highly sensitive and specific MF test is critical, for example, when microfilaria are recovered but heartworm antigen testing is negative. Some scenarios in which this might occur include death of adult worms and persistence of MF, antigen–antibody complexing precluding antigen detection, transplacental transmission of MF, or transmission of MF via transfusion [10]. Alternatively, the microfilariae may belong to a filariid species other than *D. immitis* including *Acanthocheilonema reconditum*, *Amphiachyris dracunculoides*, or *Dirofilaria repens* [12]. However, infection with the latter two species are thought to be relatively rare in the USA; these agents are more common in other parts of the world. Conversely, infection with other filariid species, such as those listed above as well as *Angiostrongylus vasorum* and *Spirocerca lupi*, which are also rare in the USA, have been reported to be cross-reactive with heartworm antigen testing, especially when immune complex-dissociation is used prior to antigen testing, resulting in falsely positive antigen tests [10, 13, 14]. The findings and discussion of this study highlight the performance of, as well as some of the advantages and limitations of, commonly used methods for the recovery of MF. Future studies interested in providing additional data regarding MF-testing would benefit from reducing time related to sampling and performance of motility-based testing, increasing the replicate number with the various testing methods, inclusion of a filtration test for comparison, and including additional levels of MF concentrations both higher and lower than the ranges worked with in this study. Ultimately, while various MF testing methods do differ somewhat in their qualitative performance and quantitative ability, an appropriate MF test should be selected in accordance with the history, signalment, and other diagnostic findings of individual patients.

## Conclusions

Microfilaria (MF) testing is an essential part of heartworm diagnosis and screening in dogs, and it is also advised by the American Heartworm Society that MF testing be performed annually for all dogs in combination with antigen testing. To our knowledge, this is the first study to directly compare the performance of eight different types of MF test. The MKT and PCR are the most qualitatively reliable tests for recovery of MF; however, clinicians should critically evaluate MF test selection on a case-by-case basis and select a test that balances practical factors, such as cost and time investment, with the patient’s risk of infection based on prevention status and history, clinical signs, and antigen testing results. This approach to MF testing will help minimize cost while avoiding failure to detect MF in infected dogs, especially when MF burden is low.

## Supplementary Information

Additional file 1. Complete data on the enumeration of microfilaria for replicates of test and sample combinations. These data were used to generate the boxplots found in Figure 1.

## Data Availability

No datasets were generated or analyzed during the current study.
