# Supplementary material for: Comparative performance analysis of different microfilaria testing methods for Dirofilaria immitis in canine blood
Source: Parasit Vectors. 2024 Nov 11;17:460. doi: 10.1186/s13071-024-06537-6 (PMC11555853; doi:10.1186/s13071-024-06537-6)
Supplement: Supplementary file 1 — Additional file 1. Complete data on the enumeration of microfilaria for replicates of test and sample combinations. These data were used to generate the boxplots found in Figure 1. [file 13071_2024_6537_MOESM1_ESM.docx]

| Sample ID | Measurement | 20 μL count | | | Wet mount | | | 9 μL HCT | | | 40 μL HCT | | | Thin smear | | | Thick smear | | | MKT | | | PCR | | |
| --- | --- | --- | --- | --- | --- | --- | --- | --- | --- | --- | --- | --- | --- | --- | --- | --- | --- | --- | --- | --- | --- | --- | --- | --- | --- |
| Dilution 1 (750 mf/mL) | Replicate counts | 18 | 12 | 13 | 31 | 24 | 25 | 1 | 3 | 2 | N/A | N/A | N/A | 2 | 4 | 1 | 18 | 17 | 7 | 299 | 321 | 311 | N/A | N/A | N/A |
|  | Average | 14.3 | | | 26.7 | | | 4.3 | | | N/A^**^ | | | 2.3 | | | 14 | | | 310.3 | | | N/A | | |
|  | Estimated mf/mL | 715.0 | | | 534.0 | | | 477.8 | | | N/A | | | 575.0 | | | 700.0 | | | 310.3 | | | N/A | | |
|  | Qualitative^*^  Outcome | Positive | | | Positive | | | Positive | | | N/A | | | Positive | | | Positive | | | Positive | | | Positive | | |
| Dilution 2 (500 mf/mL) | Replicate counts | 11 | 14 | 7 | 24 | 28 | 33 | 0 | 1 | 1 | 6 | 8 | 5 | 0 | 1 | 2 | 18 | 27 | 29 | 481 | 329 | 681 | N/A | N/A | N/A |
|  | Average | 10.7 | | | 28.3 | | | 0.7 | | | 6.3 | | | 1.0 | | | 24.7 | | | 497.0 | | | N/A | | |
|  | Estimated mf/mL | 533.3 | | | 566.7 | | | 77.7 | | | 157.5 | | | 250.0 | | | 1,235.0 | | | 497.0 | | | N/A | | |
|  | Qualitative outcome | Positive | | | Positive | | | unreliable | | | Positive | | | Positive | | | Positive | | | Positive | | | Positive | | |
| Dilution 3 (100 mf/mL) | Replicate counts | 3 | 3 | 3 | 6 | 6 | 4 | 0 | 1 | 0 | 1 | 2 | 1 | 2 | 2 | 0 | 5 | 3 | 3 | 111 | 93 | 83 | N/A | N/A | N/A |
|  | Average | 3.0 | | | 5.3 | | | 0.33 | | | 1.3 | | | 1.3 | | | 3.7 | | | 95.7 | | | N/A | | |
|  | Estimated mf/mL | 150.0 | | | 106.0 | | | 37.0 | | | 32.5 | | | 325.0 | | | 185.0 | | | 95.7 | | | N/A | | |
|  | Qualitative  Outcome | Positive | | | Positive | | | Unreliable | | | Positive | | | Unreliable | | | Positive | | | Positive | | | Positive | | |
| Dilution 4  (50 mf/mL) | Replicate counts | 1 | 2 | 1 | 3 | 1 | 1 | 0 | 0 | 0 | 0 | 1 | 3 | 1 | 0 | 0 | 0 | 3 | 1 | 21 | 19 | 15 | N/A | N/A | N/A |
|  | Average | 1.3 | | | 1.7 | | | 0 | | | 1.3 | | | 0.3 | | | 1.3 | | | 18.3 | | | N/A | | |
|  | Estimated mf/mL | 66.7 | | | 33.3 | | | 0 | | | 32.5 | | | 75.0 | | | 65.0 | | | 18.3 | | | N/A | | |
|  | Qualitative  Outcome | Positive | | | Positive | | | Negative | | | Unreliable | | | Unreliable | | | Unreliable | | | Positive | | | Positive | | |
| Dilution 5  (25 mf/mL) | Replicate counts | 2 | 0 | 0 | 1 | 0 | 1 | 0 | 0 | 0 | 0 | 1 | 0 | 0 | 0 | 0 | 0 | 0 | 1 | 21 | 24 | 13 | N/A | N/A | N/A |
|  | Average | 0.7 | | | 0.7 | | | 0 | | | 0.3 | | | 0 | | | .3 | | | 19.3 | | | N/A | | |
|  | Estimated mf/mL | 35.0 | | | 14.0 | | | 0 | | | 5.7 | | | 0 | | | 5.7 | | | 19.3 | | | N/A | | |
|  | Qualitative  outcome | Unreliable | | | Unreliable | | | Negative | | | Unreliable | | | Negative | | | Unreliable | | | Positive | | | Positive | | |
